# Supplementary material for: Ocean precursors to the extreme Atlantic 2017 hurricane season
Source: Nat Commun. 2019 Feb 22;10:896. doi: 10.1038/s41467-019-08496-4 (PMC6384944; doi:10.1038/s41467-019-08496-4)
Supplement: Supplementary file 1 — Supplementary Information [file 41467_2019_8496_MOESM1_ESM.pdf]

Ocean precursors to the extreme Atlantic 2017 hurricane season
Hallam et al.
Supplementary Data

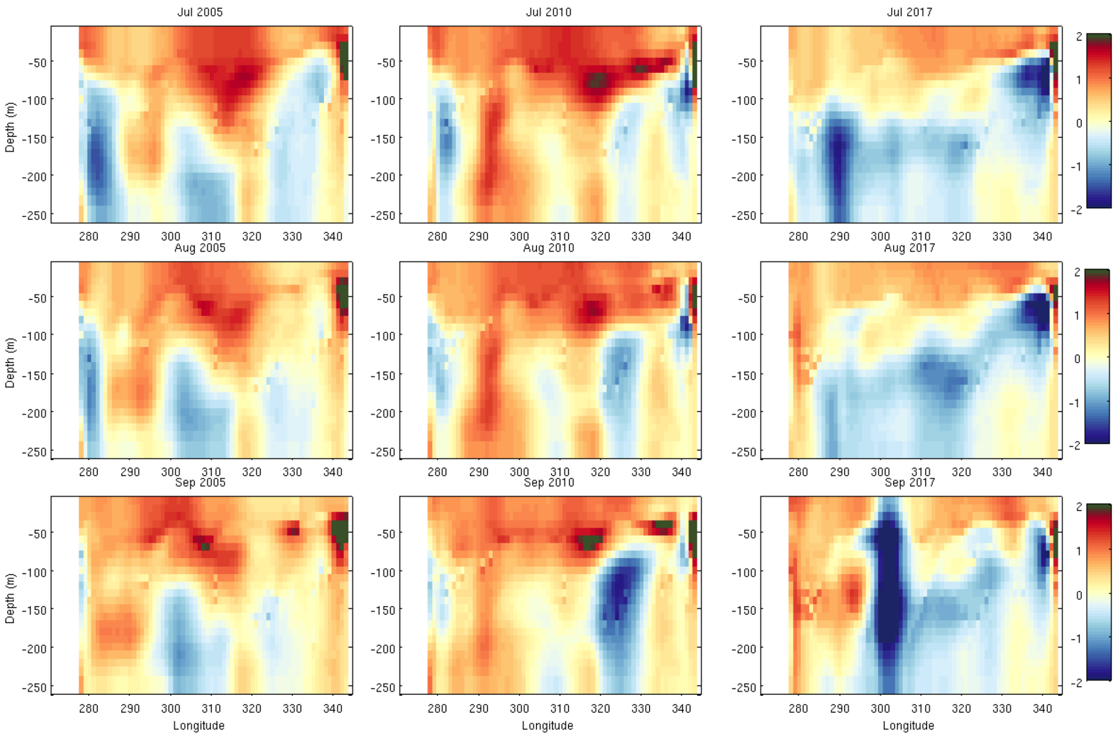

Supplementary Figure 1. Sea temperature anomalies (°C) for July – September for 2005, 2010 and 2017 between 0 - 250m in the North Atlantic at 15N from 280-350° (80°W - 10°W)

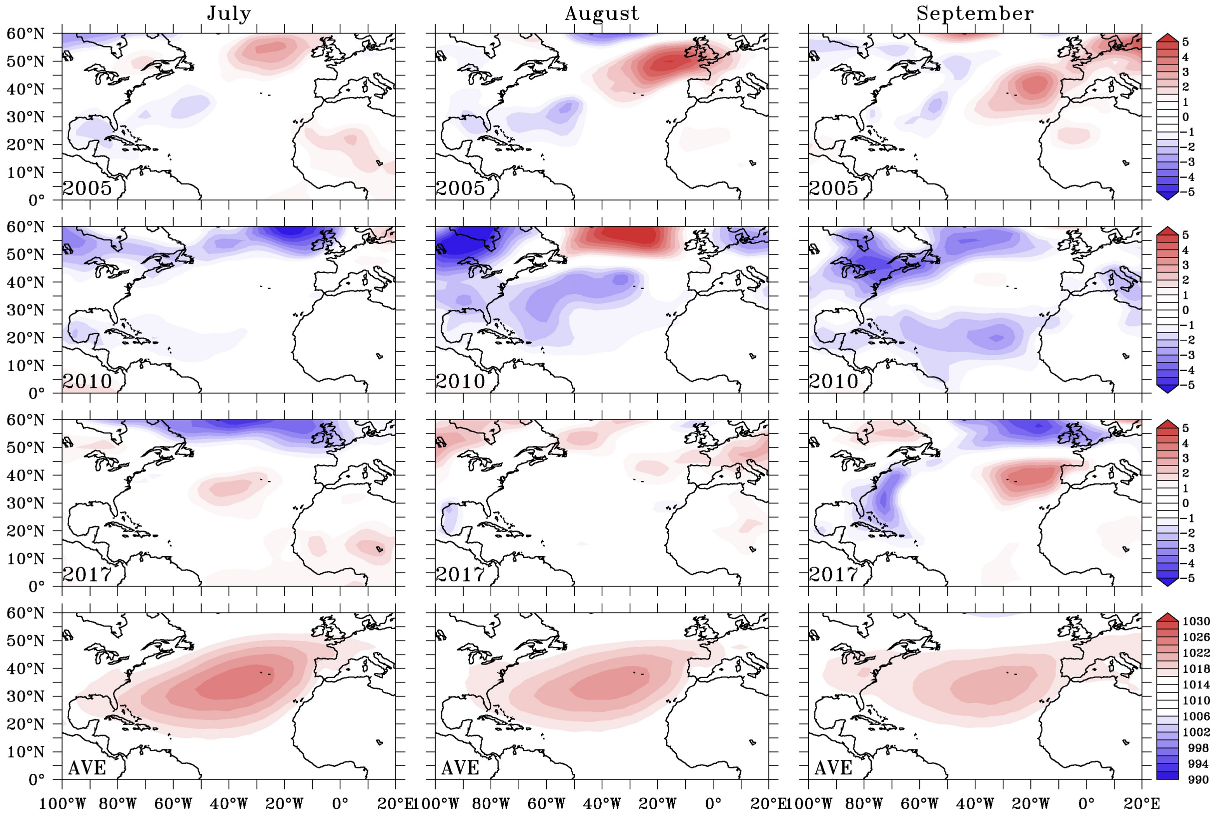

Supplementary Figure 2. Sea level pressure anomalies (mb) from July- September for 2005, 2010 and 2017 and the average climatology.

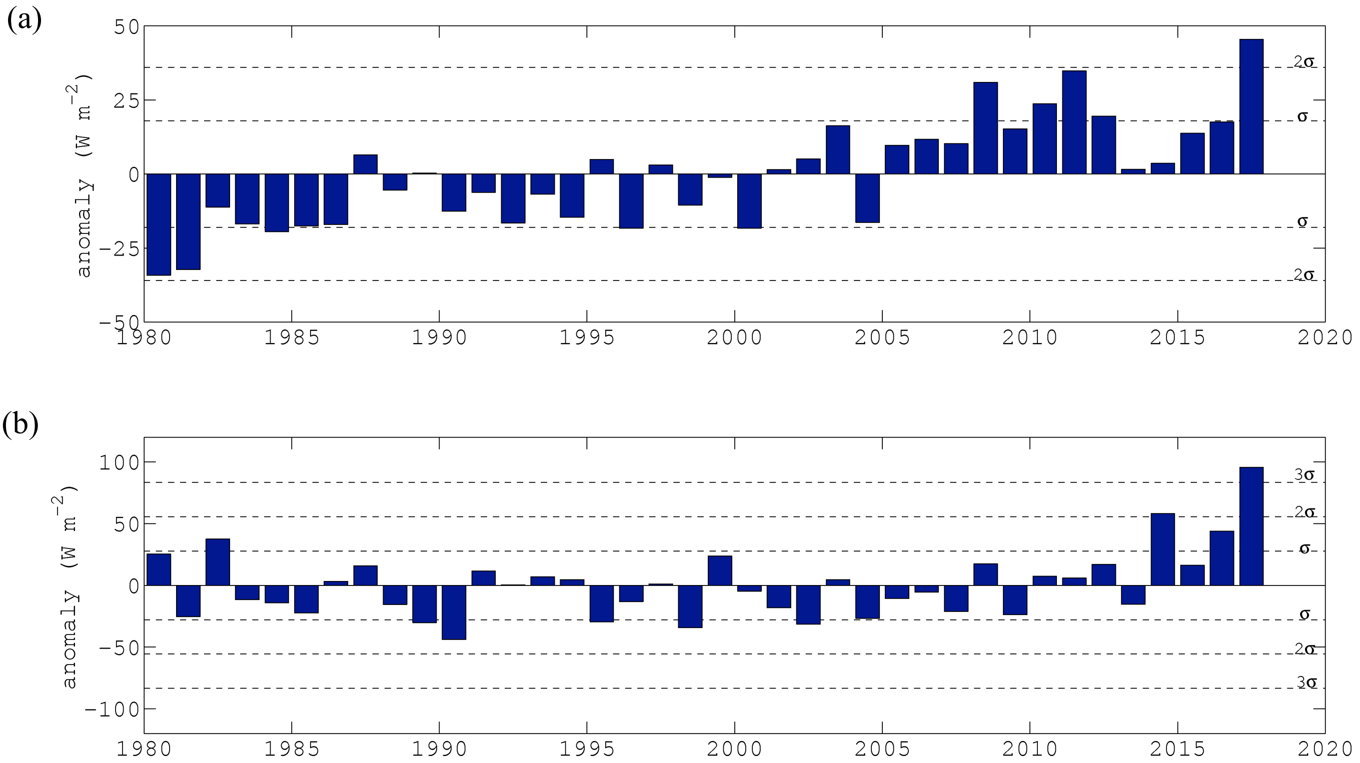

Supplementary Figure 3. Net surface heat flux anomaly timeseries (a) Net surface heat flux anomalies in the southern MDR (10-15°N, 40-60°W) from May to August for the period 1980-2017, (b) Net surface heat flux anomalies in the NE MDR (15-21°N, 24-36°W) during April for the period 1980-2017. Black dash line indicates standard deviations

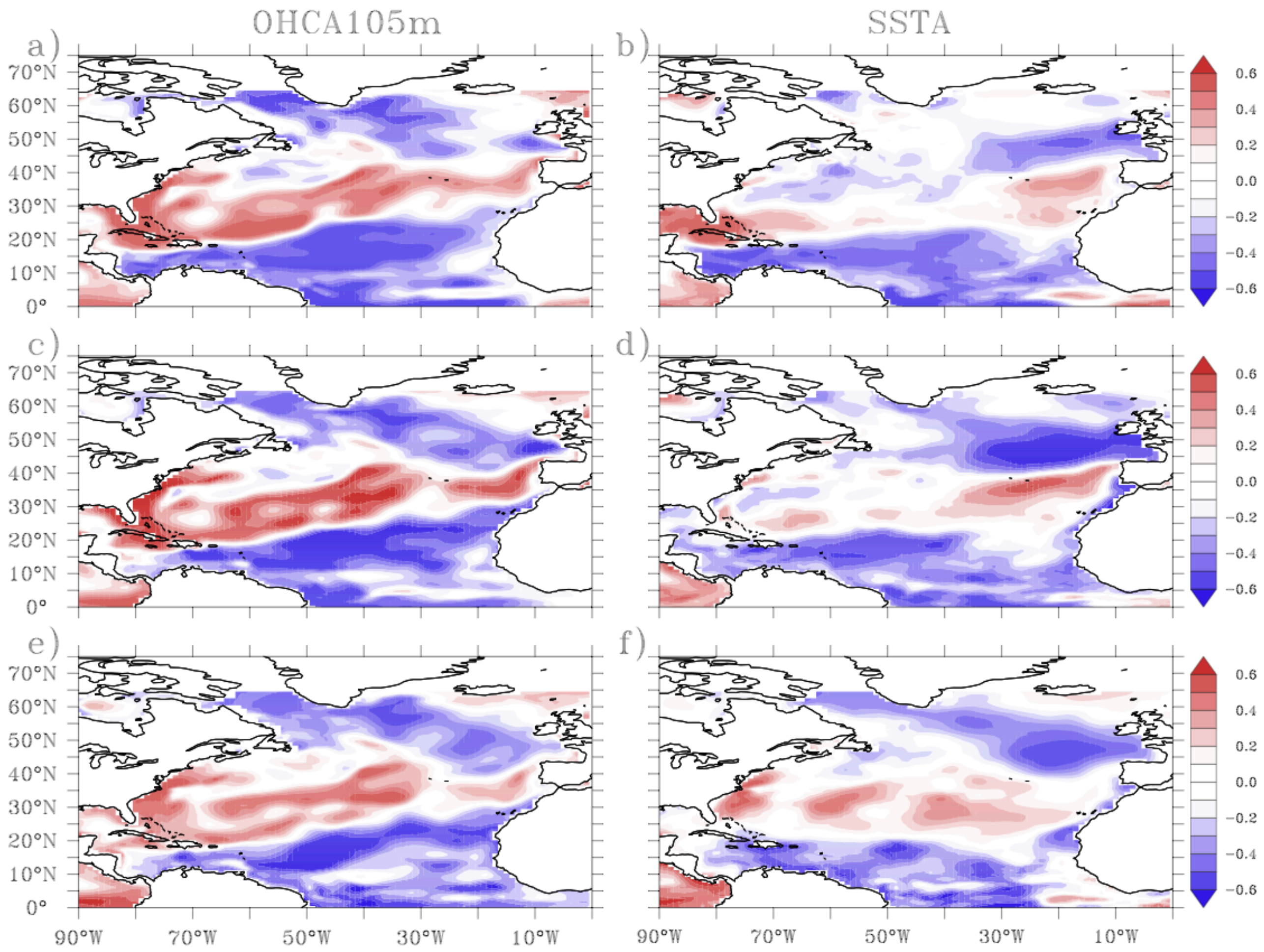

Supplementary Figure 4. Correlation between MOC anomaly during February and March and OHCA105m/SSTA in July (a - b), August (c -d), September (e -f). Correlations over 0.5 and below -0.5 are significant at the 0.05 level ($p<0.05$)

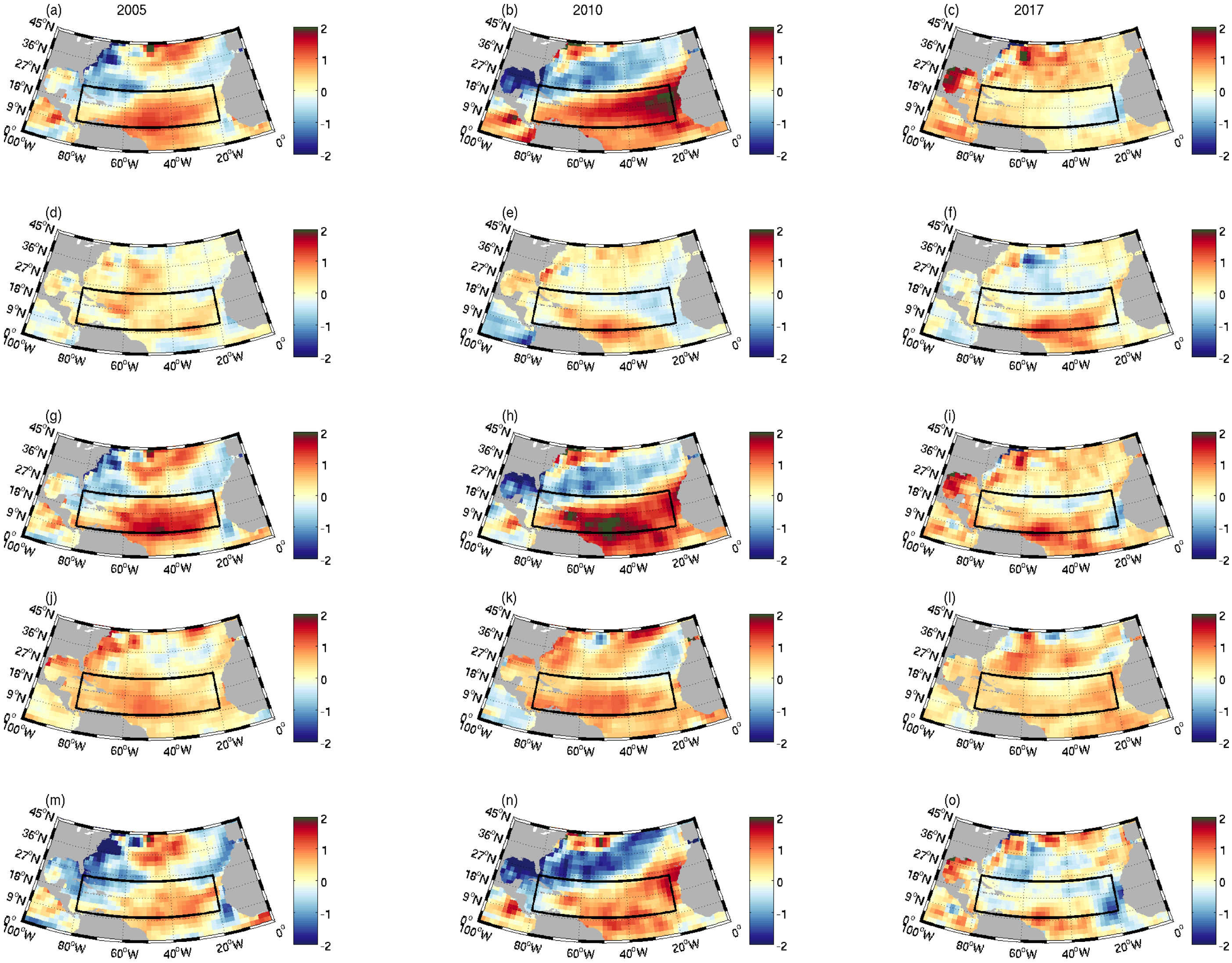

Supplementary Figure 5. Surface flux generated temperature anomaly April – July for 2005, 2010 and 2017. Initial condition – SSTA March using NCEP SST data (a - c). Estimated temperature anomaly April – July based on anomalous surface fluxes (d - f). Estimated SSTA in August formed by summing the initial condition and April – July surface flux generated temperature anomaly (g - i). Observed SSTA in August using NCEP SST data (j - l). Estimated minus observed August SSTA (m - o). Black box indicates MDR region.
